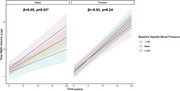# Sex‐specific associations between blood pressure and white matter hyperintensity accumulation in functionally intact older adults

**DOI:** 10.1002/alz70856_101229

**Published:** 2025-12-25

**Authors:** Anna M. VandeBunte, Emily W. Paolillo, Rowan Saloner, Molly B Memel, Molly Olzinski, Claire J. Cadwallader, Valentina E. Diaz, Coty Chen, Joel H Kramer, Kaitlin B Casaletto

**Affiliations:** ^1^ Memory and Aging Center, UCSF Weill Institute for Neurosciences, University of California, San Francisco, San Francisco, CA, USA; ^2^ Memory and Aging Center, Department of Neurology, Weill Institute for Neurosciences, University of California, San Francisco, San Francisco, CA, USA

## Abstract

**Background:**

Cardiovascular risk profiles (e.g. stroke, hypertension) differ by sex and shift across the lifespan. While males show higher risk at younger ages, postmenopausal females experience a substantial increase in vascular risk, potentially exceeding older adult males. Increasing data suggest older females also exhibit higher white matter hyperintensity (WMH) burden compared to males. Given the strong link between cardiovascular health and WMH and observed sex differences on both factors, we examined sex differences in the association between cardiovascular risk and WMH accumulation.

**Method:**

262 functionally intact older adults (Mean age=70.5; 56% female sex; CDR=0) completed baseline in‐lab blood pressure quantification (systolic blood pressure [BP]), a key marker of cardiovascular health, and at least two longitudinal neuroimaging visits (log‐transformed total WMH volume, total gray matter volume [GMV]). Total number of annual visits ranged from 2‐7. Linear mixed‐effects models evaluated whether WMH trajectories over time differed by sex. Next, interaction models tested whether longitudinal associations between baseline systolic BP and WMH trajectories differed by sex. To test the specificity of these effects to white matter, we examined the same models with total GMV as the outcome. All models adjusted for baseline age and included person‐specific random intercepts and slopes.

**Result:**

Baseline systolic BP and WMH volume did not differ by sex (*p*s>0.05). Longitudinal WMH trajectories differed by sex (β=‐0.10, *p* = 0.002), however, such that females showed steeper accumulation of WMHs over time. Baseline systolic BP did not significantly associate with WMH trajectories (β=0.01, *p* = 0.67); however, this association differed by sex (β=‐0.08, *p* = 0.01) such that higher baseline systolic BP associated with increasing WMH burden over time in males only (Figure 1). In specificity models, greater baseline systolic BP associated with steeper GMV loss over time (β=‐0.03, *p* = 0.006); however, this did not differ by sex (β=0.00, *p* = 0.083).

**Conclusion:**

Functionally intact females show greater WMH accumulation over time compared to males. However, systolic BP was a significant predictor of WMH burden in males only, suggesting other factors may contribute to female WMH burden. While higher systolic BP associated with GMV loss over time, sex differences appeared specific to WMH. These findings underscore the importance of considering sex‐specific mechanisms of vascular contributions to brain aging.